# The role of exosomal PD-L1 in tumor progression and immunotherapy

**DOI:** 10.1186/s12943-019-1074-3

**Published:** 2019-10-23

**Authors:** Feiting Xie, Mengxue Xu, Jian Lu, Lingxiang Mao, Shengjun Wang

**Affiliations:** 1 Department of Laboratory Medicine, The Affiliated People’s Hospital, Jiangsu University, Zhenjiang, 212013 China; 2 Department of Immunology, Jiangsu Key Laboratory of Laboratory Medicine, School of Medicine, Jiangsu University, Zhenjiang, China

**Keywords:** Exosomes, PD-L1, PD-1, Tumor immunity, Biomarker

## Abstract

Programmed death ligand 1 (PD-L1), a type I transmembrane protein, binds to its receptor PD-1 to suppress the activation of T cells, thereby maintaining immunological homeostasis. In contrast, tumor cells highly express PD-L1, which binds to receptor PD-1 expressed on activated T cells, leading to immune escape. Anti-PD-1/PD-L1 immune checkpoint therapy blocks the binding of PD-1/PD-L1 to reinvigorate the exhausted T cells, thereby inhibiting tumor growth. Exosomes are biologically active lipid-bilayer nanovesicles secreted by various cell types that mediate intercellular signal communication. Numerous studies have shown that tumor cells are able to promote tumor epithelial-mesenchymal transition, angiogenesis, and immune escape by releasing exosomes. Recent studies imply that tumor-derived exosomes could carry PD-L1 in the same membrane topology as the cell surface, thereby resisting immune checkpoint therapy. In this review, we mainly discuss the role of exosomes in the regulation of tumor progression and the potential resistance mechanism to immunotherapy via exosomal PD-L1. In addition, we propose that exosomal PD-L1 may have the potential to be a target to overcome resistance to anti-PD-1/PD-L1 antibody therapy.

## Background

Programmed death ligand 1 (PD-L1), also known as cluster of differentiation 274 (CD274) or B7 homolog (B7 homolog 1, B7-H1), is a type I transmembrane protein of 290 amino acids encoded by the CD274 gene and consisting of immunoglobulin V-like and C-like extracellular domains [1]. PD-L1 is widely expressed on various cell types, mainly in tumor cells, monocytes, macrophages, natural killer (NK) cells, dendritic cells (DCs), and activated T cells and also on immune-privileged sites such as the brain, cornea, and retina [2]. In normal physiological conditions, the activation of the PD-1/PD-L1 signaling pathway is closely related to the induction and maintenance of peripheral tolerance, maintenance of T cell immune homeostasis, avoiding hyperactivation and protecting against immune-mediated tissue damage [3]. In disease states, PD-L1 interacts with its receptor programmed death 1 (PD-1), transmitting a negative signal to control a series of processes of T cell-mediated cellular immune responses, including priming, growth, proliferation and apoptosis, and functional maturation [4]. Recently, research studies have found that the activation of the PD-1/PD-L1 signaling pathway can arrest the T cell cycle at the G1 phase rather than directly causing apoptosis [5]. In addition to mediating T cell–intrinsic inhibitory effects, the PD-1/PD-L1 signaling pathway could also induce the inhibition of T cell responses by inducible Tregs (iTregs) [6].

Recent studies on extracellular vesicles have demonstrated that exosomes are biologically active lipid-bilayer nanovesicles (30–120 nm) secreted by almost all types of normal or diseased cells through endosomal pathways into the extracellular space to transport “cargo” to the target cells to mediate intercellular signal communication [7–10]. The “cargo” is mainly composed of cytosolic proteins, microRNAs, circular RNAs, long noncoding RNAs, lipids, DNA, cytokines, transcription factor receptors and other molecules [11]. Research has shown that normal or diseased cells can secrete exosomal miRNAs to affect neighboring cells and/or distant target cells [12]. Noncoding RNAs in exosomes could influence the expression of tumor-related genes to cooperate in the cancer-causing process [13]. A rapidly growing number of studies have shown that exosomes participate in multiple physiological and pathological processes, especially the development of cancers.

Exosomes are widely distributed in body fluids such as saliva, plasma, urine, breast milk, and amniotic fluid both in cancer and noncancer cells from tumor patients [14]. Emerging evidence has shown that the exosomes secreted by tumor cells carry bioactive PD-L1 on their surface and can suppress the immune response [15]. Metastatic melanoma-derived exosomes, which are stimulated by interferon-γ (IFN-γ), expressed more PD-L1 on these vesicles and inhibited antitumor responses [16]. In this review, we mainly discuss the role of exosomes in the regulation of tumor immunity and the potential resistance mechanism to immunotherapy via exosomes that express PD-L1 on their surface (the same membrane topology as cell surface PD-L1).

## The regulatory role of exosomes in tumor progression

### Exosomes inhibit tumor progression

In the tumor microenvironment, exosomes may mediate intracellular communication and immune regulation. Tumor-derived exosomes (TEXs) and immune cell-derived exosomes (IEXs) have been demonstrated to activate immune responses by transferring antigens to antigen presenting cells (APCs), which results in the activation of CD4^+^ T cells and CD8^+^ T cells, thereby enhancing the anti-tumor responses and leading to the inhibition of tumor progression [17]. Additionally, an alternative mode of action has also been described that involves inducing the apoptosis of tumor cells [18]. The latest research has found that TEXs and IEXs could be applied to oncotherapy as monitoring markers instead of detecting T lymphocytes and taking tumor biopsies [19]. In the following description, we introduce the anti-tumor effect of TEXs and IEXs. (Tables 1 and 2).

**Table 1:** The regulatory role of tumor cell-derived exosomes in tumor progression

Origin of Exosomes	Target cells							Tumor progression	References
	DC	CTL	Treg	Th17	M2	NK	Monocyte		
Heat-stressed tumor cell	↑		↓	↑				↓	[20]
Hepatocellular carcinoma cell	↑	↑↓			↑	↓		↑↓	[21–24]
Malignant glioma cell	↑	↑						↓	[25]
Leukemia cell	↑	↑				↑		↓	[26, 27]
RenCa cell		↑						↓	[28]
Gastric cancer cell		↓					↑	↑	[29]
Melanoma cell		↓						↑	[16, 30, 31]

Tumor progression includes: ↑promotion, ↓inhibition

**Table 2:** The regulatory role of immune cell-derived exosomes in tumor progression

Origin of Exosomes	Target cells					Tumor progression	References
	DC	CD4^+^ T	CTL	Treg	Th17		
DC		↑	↑	↓		↓	[32]
CD4^+^ T cell			↓			↑	[33]
CD8^+^ T cell	↓		↑			↑↓	[7, 34]
Macrophage		↑				↓	[35]
TAM	↓			↑	↑	↑	[32]
Mast cell	↑	↑	↑			↓	[35]
B cell	↑	↑	↓↑			↓↑	[35, 36]
MDSC			↓			↑	[37]
Treg			↓			↑	[38]

Tumor progression includes: ↑promotion, ↓inhibition

*DC* Dendritic cell, *MSC* Mesenchymal stem cell, *CTL* Cytotoxic T lymphocyte, *NK* Natural killer, *M2* M2 macrophage, *TAM* Tumor-associated macrophages cell, *Treg* Regulatory T cell, *MDSC* Myeloid-derived suppressor cell, *Th* T helper

Considering the origin of exosomes, TEXs may contain some tumor-associated antigens, including melan A, carcinoembryonic antigen and mesothelin [39, 40]. Thus, TEXs could be used to form a pool of tumor antigens to stimulate the anti-tumor response. Currently, TEXs have been widely used for the induction of anti-tumor responses in both murine models and clinical trials. A recent study reported that exosomes derived from heat-stressed tumor cells could induce the production of IL-6 by DCs and marcophage, which switches regulatory T cell into Th17 in tumor microenviroment in a HSP-70 dependent manner [20]. DCs have been proven to be a target for TEXs to enhance anti-tumor responses [21]. Research has found that EG7 tumor cell-derived exosomes transfer parental cell-associated antigen OVA and pMHC-I to DCs, which stimulate a stronger proliferation and differentiation of cytotoxic T lymphocytes (CTL) and generating a more robust OVA-specific antitumor immunity than control ones. Similar results were obtained in hepatocellular carcinoma (HCC) models and in other studies [21, 22]. Simultaneously, exosomes from TGF-β-silenced leukemia cells decrease the secretion of TGF-β by DCs and effectively promote their maturation and function. Additionally, DCs carrying these exosomes facilitated the proliferation of CD4^+^ T cells and enhanced the antigen-specific CTL responses [26, 27]. Interestingly, TEXs which exert a stable antitumor response are mostly based on targeting DCs. These provide a new idea for our future research.

It has been reported that IEXs also contribute to enhancing the anti-tumor response. In addition, IEXs could alter the microenvironment suitable for tumors to suppress tumor growth. Recently, DC-derived exosomes (DEXs) have been recognized as a new class of vaccines for tumor therapy [35, 41]. In this research, Lu and coworkers found that exosomes derived from a-fetoprotein (AFP)-expressing DCs could promote the antigen-specific immune response through elevating the levels of IFN-γ and interleukin-2 and reducing the expression of interleukin-10 and TGF-β. Activated CD8^+^ T cell-derived extracellular vesicles are able to directly target mesenchymal tumor stromal cells to prevent tumor invasion and metastasis [34]. Exosomes released by NK cells have also been identified as having therapeutic effects. Both in vitro and in vivo experiments revealed that NK cell-derived exosomes could suppress the development of melanoma via their contents of TNF-α, perforin and FasL [42]. In neuroblastoma (NB) tumors, exosomes derived from NK cells pretreated with NB cells increased the expression of natural killer cell receptors and enhanced the cytotoxicity of NK cells against NB tumors [43].

In addition to the exosomes mentioned above, exosomes derived from mesenchymal stem cells (MSCs) have also been reported to restrain tumor development [44]. MSC-derived exosomes have potent regulatory effects on immune responses involving different immune cells, such as T cells and B cells [45]. Researchers have demonstrated that human adipose MSC-derived exosomes inhibit the proliferation and colony formation ability of A2780 and SKOV-3 human ovarian cancer cells via inducing the expression of BAX and CASP3/9 while reducing the levels of BCL2 [46]. Interestingly, researchers have obtained similar results from human umbilical cord MSC-derived extracellular vesicles (EVs) [47].

### Exosomes promote tumor progression

Despite exosomes having anti-tumor effects as mentioned above, more studies have focused on their effects in promoting tumor progression [48]. The growth of tumor is associated with various growth factor receptors and signaling pathways. These receptors triggers downstream signaling pathways via Akt, PKC/PKB and ERK kinase pathways through the activation or phosphorylation of intracellular kinase domain which leads to tumor cell proliferation and migration.

In NB tumors, TEXs promote tumor proliferation and migration by decreasing the expression of NEDD4 by hsa-miR199a-3p [49]. TEXs derived from the Lewis lung cancer model and 4 T1 breast cancer model inhibit the differentiation of myeloid precursors into DCs and their maturation by increasing the expression of immunosuppressive markers PD-L1 to trigger inhibitor signals [50]. Moreover, studies have reported that gastric cancer-derived exosomes can induce monocytes to differentiate into PD-1^+^ tumor-associated macrophages (TAMs), which can effectively suppress anti-tumor responses by triggering the PD-1/PD-Ls signaling pathway [51]. Exosomes derived from oral squamous cell carcinoma could upregulate the expression of PD-L1 on myeloid-derived suppressor cells (MDSCs) to induce ɣδ T cell exhaustion through an exosome miR-21/PTEN/PD-L1 pathway [52]. Similar results have been obtained in hepatocellular carcinoma (HCC) cells. Liu J and colleagues discovered that exosomes released from HCC cells upregulated the expression of PD-L1 in macrophages to inhibit T-cell function through a miR-23a/PTEN/AKT regulation axis [53]. In addition, exosomes obtained from melatonin-treated hepatocellular carcinoma cells were able to downregulate the expression of PD-L1 and the secretion of cytokines (IL-6, IL-1β, IL-10, and TNF-α) in macrophages [54]. In light of this, we surmise that miRNAs contained in tumor-derived exosomes may facilitate the growth and metastasis of tumors [55].

IEXs and exosomes secreted by other cells have also been reported to facilitate the growth and metastasis of tumors. Exosomes derived from activated CD8^+^ T cells can promote the invasion of tumor cells via the Fas/FasL pathway [7]. However, another study has shown that activated T cell-derived exosomes promote tumor invasion and metastasis through the ERK and NF-κB pathways. Exosomes released by activated OVA-specific CD4^+^ T cells can suppress the cytotoxicity of DC-stimulated CD8^+^ T cells via pMHC II/TCR and CD54/LFA-1 interactions. Recently, a study described the functions of EVs derived from B cells in anti-tumor responses [36]. CD39^+^CD73^+^ EVs released by tumor B cells hydrolyze ATP and AMP from tumor cells into adenosine to inhibit the proliferation of CD8^+^ T cells. In a clinical trial, higher CD19^+^ EVs in tumor patients’ serum were associated with poor efficacy of chemotherapy. These results indicate that exosomes may not have the characteristics of the parent cells [33]. In osteosarcoma and gastric cancer patients, researchers have found that human bone marrow MSC-derived exosomes (MSC-exo) could promote tumor progression through the activation of the Hedgehog signaling pathway [56]. Our data shown that exosomal S100A9 from granulocytic myeloid-derived suppressor cells promotes CRC cell stemness and growth [9].

Whether pro-tumor or anti-tumor function of TEXs and IEXs, these researches state the importance of them in tumor progression and define emergence of new paradigms in tumor cell biology. In terms of tumor inhibition, IEXs exert anti-tumor response primarily through their contents, such as TNF-α, perforin, FasL and so forth. However, TEXs activate the anti-tumor immune response mainly by presenting tumor antigens to DCs. In terms of tumor promotion, the molecules expressed on IEXs, such as FasL and pMHC II, interact with T cells to trigger co-inhibitor signals and promote apoptosis. TEXs have the similar effect as IEXs. The contents contained in TEXs have the ability to up-regulate the expression of PD-L1 on myeloid cells to inhibit the function of T cells through PD-1/PD-L1 signaling pathway. And exosomal MHC I could promote this inhibit function by inducing the first signal activation of T cells. Recent studies have demonstrated that exosomes can carry PD-L1 on their surface and have been proven to play a key role in tumor development [57–59]. Because of their remarkable immunosuppressive function, exosomes may be a cancer therapy target in the future.

## Upregulated expression of PD-L1 induces immune escape to promote tumor development

### The function of PD-L1 expressed on cells in suppressing anti-tumor immune response

The expression of PD-L1 is upregulated in different types of cancer and noncancer cells, such as macrophages, monocytes, and tumor cells. Its receptor PD-1 is mainly expressed on activated CD4^+^ and CD8^+^ T cells, B cells, monocytes and MDSCs [60]. One of the important mechanisms of tumor immune evasion is that PD-L1 expressed on the tumor cell surface binds to PD-1 on T cells, leading to an immune checkpoint response. Under normal conditions, activated antigen-specific cytotoxic T lymphocytes can recognize and directly respond to tumor cells. However, PD-L1-overexpressing tumor cells have the exceptional capability to survive, escape the immune system surveillance and then invade neighboring tissue [61].

In recent clinical studies, researchers have found that the high expression of PD-L1 in patients with triple-negative breast cancer tissue may be associated with poor prognosis [62]. Recent studies have found that tumor cells have the potential to control the molecular mechanism (depletion of HIP1R) of lysosomal degradation of PD-L1, leading to its intracellular accumulation and inducing immune tolerance. The peptide PD-LYSO successfully targets PD-L1 expressed by tumor cells to lysosomes for degradation, taking the place of the depleted HIP1R [63]. Moreover, Chen J. et al. demonstrated that IFN-γ, which is secreted by inflammatory cells such as macrophages and NK cells, can upregulate PD-L1 expression on tumor cells [64, 65]. With upregulated expression of PD-L1, tumor cells mediate adaptive resistance to the IFN-γ released by cytotoxic T lymphocytes, creating a vicious circle that exacerbates the disease [65]. In gastric cancers, several previous reports have demonstrated that PD-L1 expression can range from 25 to 65% on tumor cells [51].

Simultaneously, studies have shown that the infiltration of immune cells in the tumor microenvironment may be associated with the presentation of PD-L1 on their surface [66]. In the tumor hypoxic microenvironment, the expression of PD-L1 on MDSCs is increased, and the changing level of PD-L1 can be used to evaluate the efficacy of hyperoxic therapy [67]. Moreover, TAMs, which overexpress PD-L1 on the cell surface, may suppress the function of cytotoxic T lymphocytes. Another hypothesis has suggested that the TAM receptors Tyro3, Axl, and Mertk could upregulate the expression of PD-L1 on a breast cancer cell line [68]. Furthermore, IFN-γ could facilitate the level of PD-L1 expressed on exosomes released by melanoma cells that we will discuss later in this review. Thus, the potential effects of PD-L1 overexpression on different cell types are not clearly understood.

### Exosomal PD-L1 is involved in inducing immune escape to promote tumor progression

A previous study verified that PD-L1 exists in exosomes from human urine or plasma [69]. Studies have confirmed that the levels of PD-L1 expressed on exosomes, but not soluble PD-L1, correlate with disease progression in head and neck squamous cell carcinoma (HNSCC) [58]. Flow cytometry and immunofluorescence results suggested that PD-L1 is present not only on the surface of vesicles but also within vesicle-like structures [70]. PD-L1 could be specifically packaged into exosomes with the help of both Rab27a and nSMase2. Knocking out Rab27a and nSMase2 in tumor cells causes a loss of PD-L1 in the secreted fraction. Researchers found that exosomal PD-L1 has the same membrane topology as cell surface PD-L1 using an enzyme-linked immunosorbent assay [16]. Recent reports have visually pointed out that exosomal PD-L1 can transfer to other cells with or without PD-L1 expression in a dose-dependent manner in vitro and in vivo [71]. As the number of studies of PD-L1 on exosomes increases, its critical role in research has been gradually revealed. It was worth mentioning that the results referred below are all about tumor-derived exosomal PD-L1, although the expression of PD-L1 on myeloid cell is critical in inducing immune suppression, there is no published report studying about the myeloid cell- derived exosomal PD-L1.

Glioblastoma, a local and systemic immunosuppressive neoplasm, is able to secrete inflammatory cytokines, initiating the immune checkpoint response. In addition, EVs released by glioblastoma stem-like cells (GSCs) express PD-L1 on their surface and play a critical role in mediating the inhibition of both CD4^+^ and CD8^+^ T cell activation and proliferation by an anti-CD3 mAb [72]. In the 4-nitroquinoline 1-oxide-induced malignant oral/esophageal injury model, exosomes carrying PD-L1 isolated from supernatants of murine or human HNSCC cell lines can hamper the infiltration of CD4^+^ T and CD8^+^ T cells into the tumor microenvironment, thereby accelerating tumor progression [73]. Exosomal PD-L1 from TRAMP-C2 cells has no significant differences between Rab27a and PD-L1 knockout phenotypes in promoting tumor progression [74]. However, in MC38 tumor models, PD-L1 knockout plays a more critical role than Rab27a loss does. Thus, in different tumor models, the role of exosomal PD-L1 in promoting tumor progression is diverse.

To address whether the loss of exosomal PD-L1 would reverse the PD-L1-mediated immunosuppression in vivo and in vitro, researchers established a xenograft model with PD-L1 knockdown cell lines and then found that tumor growth was promoted by the exosomes released by parental cells. The same conclusion has been reached in 4 T1-PD-L1 (knockdown) tumor models. In the draining lymph nodes of mice injected with Rab27a-null or PD-L1-null TRAMP-C2 cells, the percentage of CD8-positive cells is upregulated, and the proportion of cells expressing the exhaustion marker Tim3 is downregulated, while the proportion of cells expressing the activation markers granzyme B and Ki67 is increased. These results indicate that the loss of exosomal PD-L1 can relieve the inhibition of T cell activation in the draining lymph nodes. Moreover, exosomal PD-L1 plays an important role in not only immediate immune response but also memory ones. Mice injected with either Pd-l1 or Rab27a null TRAMP-C2 cells on one flank not only suppresses growth of the local tumor cells but also blocks WT tumor cells rechallenged on the other flank in 90 days. This indicated that T cells derived from a xenograft model lacking exosomal PD-L1 possess the ability to exert a robust antitumor memory response resistant to the inhibitory effect of exosomal PD-L1. In addition, exosomal PD-L1 derived from other cancer cells such as colon and lung cancer cells has similar effects on T cell activation and tumor progression. (Table 3).

**Table 3:** The function of exosomal PD-L1 in tumor progression

Type of tumor	Source of exosomes	Function	References
Melanoma	Plasma	Suppress the function of CD8^+^ T cells and cause failure of anti-PD-1 therapy	[16]
Breast cancer	Tumor tissue	Inhibit the secretion of granzyme B by cytotoxic lymphocytes to promote tumor growth	[71]
Prostate cancer	Tumor tissue	Suppress the function of T cells in the draining lymph node and block anti-PD-L1 antibodies	[70]
Head and neck squamous cell carcinomas	Plasma	Downregulate CD69 expression on effector T cells to inhibit anti-tumor responses	[58]

A recent study roughly divided solid tumors into ‘hot’ or ‘cold’ according to whether the tumors were surrounded by T cells or not [75–77]. In view of the function of exosomal PD-L1 mentioned above, we suspect that PD-L1 may have an effect on T cells migrating to the tumor site.

### Exosomal PD-L1 mediates resistance to immunotherapy by directly binding to anti-PD-L1 antibody

In the past few decades, the immunotherapy of tumors has captured widespread attention in the medical community. Immunotherapy can be broadly divided into two different types: somatic immunotherapy and immune checkpoint inhibitor treatment. Immune checkpoint blockade treatments include anti-CTLA-4 mAb, anti-PD-1 mAb, and anti-PD-L1 mAb [78]. It has been reported that the attractive system E1A-engineered mesenchymal stromal cell (MSC. E1A) can locally modify tumor cells to release CD3-HAC (PD-1), which can be bound to PD-L1-positive breast cancer cells. Therefore, the recruited T cells can exert their potential role in anti-tumor immunity [79]. A study has demonstrated that monoclonal antibodies against PD-1/PD-L1 demonstrate prominent effectiveness in patients with different types of cancers, such as non-small-cell lung cancer (NSCLC), HNSCC, melanoma, colorectal cancer, and breast cancer [80–84]. However, some solid tumors, such as prostate cancer, have been shown to be resistant to the treatment with anti-PD-L1 therapy [85]. The mechanism of therapeutic resistance among patients remains largely unknown. In view of the roles of exosomal PD-L1 in tumor progression, we asked whether exosomal PD-L1 could induce therapeutic resistance to anti-PD-L1 antibody treatment.

It is widely known that PD-1/PD-L1 blockade could activate T cells, but little is known about the role of exosomal PD-L1 in the relatively low response rate of anti-PD-L1/PD-1 therapy [86]. Pretreatment of glioblastoma EVs with anti-PD-1 receptor-blocking antibodies nearly reversed the suppression of T cells and prevented tumor progression [72]. Chen and colleagues have also found that PD-L1 on metastatic melanoma-derived exosomes inhibits the activation of CD8^+^ T cells and facilitates tumor growth, and these effects can be disrupted by anti-PD-1 antibody (aPD-1) therapy [16]. In a prostate cancer syngeneic model, mice were not responsive to anti-PD-L1 antibody (aPD-L1) treatment because of exosomal PD-L1. In 4 T1 tumor models, the accumulation of exosomal PD-L1 in the tumor microenvironment induced immunotherapy resistance by suppressing the secretion of granzyme B. Notably, knockdown of Rab27a in tumor cells significantly enhanced the efficiency of anti-PD-1 therapy and inhibited 4 T1 tumor growth [71]. In HNSCC patients, PD-L1^high^ exosomes significantly inhibit CD69 on CD8^+^ T cells and can be blocked by anti-PD-1 antibodies [58]. These results indicate that exosomal PD-L1 plays an important role in anti-PD-L1/PD-1 therapy. However, the concrete resistance mechanism of exosomal PD-L1 is largely unclear. Not only exosomal PD-L1 but also PD-L1 splicing variants play a crucial role in tumor progression. Cells from patients who secreted PD-L1 splicing variants were able to competitively bind to aPD-L1 antibody, which lead to therapeutic resistance [87]. Recent data implied that tumor- and regulatory T cell (Treg)-derived exosomes carrying CTLA-4 on their surface could interfere with immunotherapy with ipilimumab. These results suggest that the molecules carried on exosomes may be a novel mechanism for patients to generate therapeutic resistance and may be potential biomarkers for tumor diagnoses [19].

**Fig. 1:**
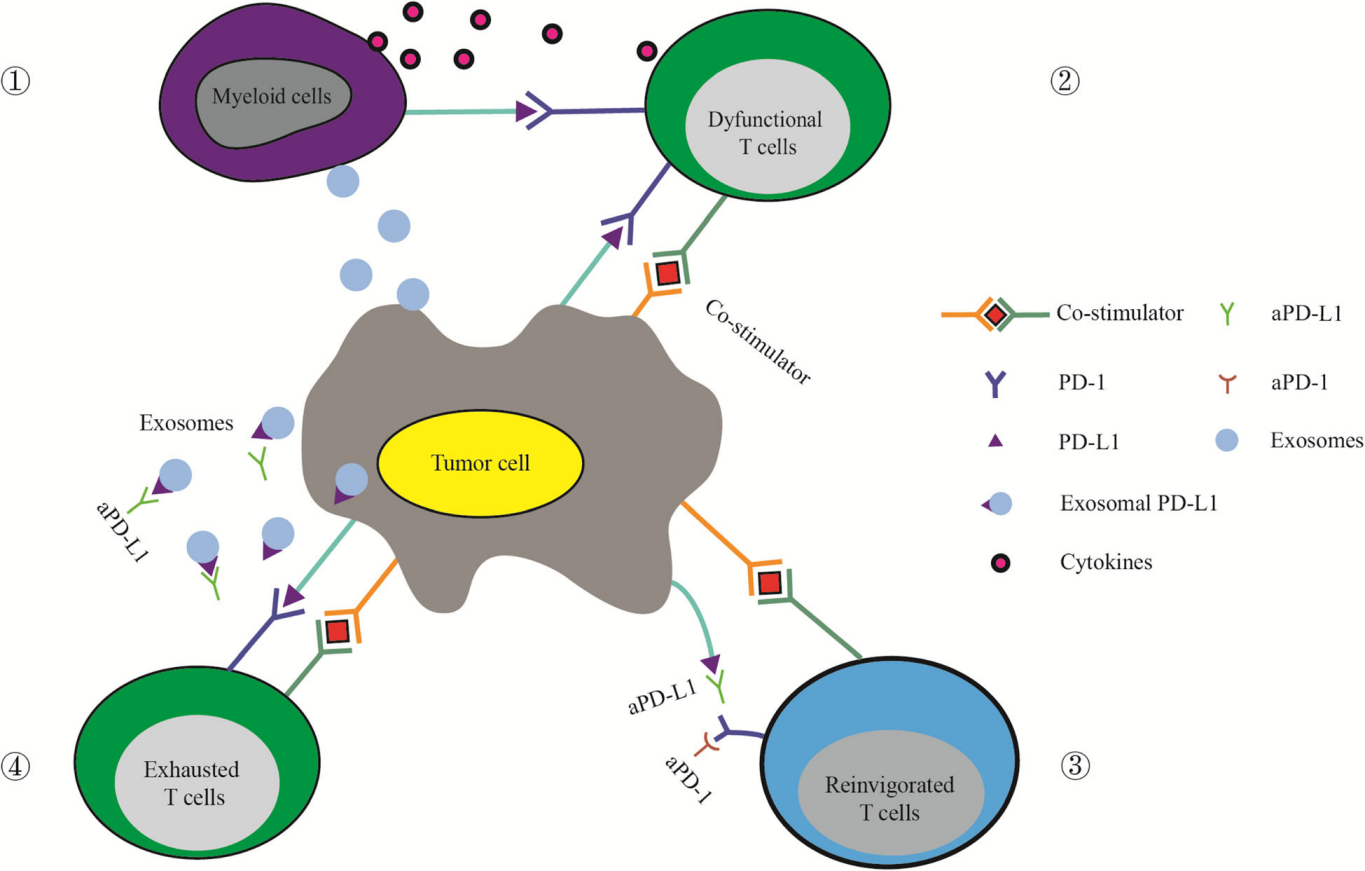
Exosomal PD-L1 contributes to the resistance to immune checkpoint therapy. ①Tumor-derived exosomes up-regulate the expression of PD-L1 and the production of cytokines by myeloid cells to inhibit the function of T cells. ② PD-L1 expressed on the tumor surface directly binds to PD-1 on T cells, inducing an immune checkpoint response. The checkpoint response can suppress the activation of T cells and make T cells dysfunctional to inhibit anti-tumor immunity. ③ Immune checkpoint therapy can free the T cells limited by checkpoint blockades to rejuvenate immune responses. ④ Exosomal PD-L1 has the same membrane topology as PD-L1 on the cell surface and could suppress the function of T cells and cause the failure of immune checkpoint therapy

Although anti-PD-L1/PD-1 therapy has already been applied to clinical studies, questions remain to be further explored. Scientists have confirmed that after sequential use of PD-1 and PD-L1 antibodies, fatal myocarditis developed in a patient with brain metastatic lung adenocarcinoma [88]. Another research study demonstrated that anti-PD-1/PD-L1 treatment may offer a potential immunotherapy to cure Alzheimer’s disease and dementia [89]. Although immune checkpoint blockade has fewer side effects than conventional chemotherapy and radiotherapy, its specific mechanism remains to be studied in detail [90]. (Fig. 1).

### Exosomal PD-L1 may act as a tumor biomarker

Prior studies have focused on the use of exosomes as a genetic engineering vaccine targeting tumor cells, but an exponentially increasing number of studies has found that tumor-derived exosomes could be tumor diagnostic markers. In the past few decades, many researchers have committed to finding biomarkers in exosomes to specifically detect or predict diseases [91].

Exosomes can express various function-related molecules that are detected on the membranes of parent cells. The prognosis of head and neck cancer or melanoma patients varies with the different levels of circulating exosomal PD-L1. Before receiving treatment, the levels of circulating exosomal PD-L1 in patients who responded to anti-PD-1 antibodies were higher than those in nonresponders. The fold change of exosomal PD-L1 was more significant in responders than nonresponders after undergoing therapy. Hence, exosomal PD-L1 may be a biomarker for clinical immunotherapy to detect its levels in patient blood prior to therapy. Another study has demonstrated that the detection of PD-L1^+^ exosomes in serum was correlated with poor prognosis in patients with pancreatic ductal adenocarcinoma [92]. Further study has implied that miR-21 contained in PD-L1^+^ exosomes has the potential to be a biomarker for distinguishing the differences between NSCLC patients and healthy controls [93]. Going forward, in the process of anti-PD-1/PD-L1 immune checkpoint therapy, we should take not only the cell surface PD-L1 but also the exosomal ones into account.

In short, the results mentioned above provide a theoretical basis for exosomal PD-L1 as a biomarker, but need to be expanded to make the liquid biopsy of exosomes possible.

## Conclusion and future perspective

All together, both tumor-derived exosomes and immune cell-derived exosomes play dual roles in tumor progression. Although exosomes could inhibit cancerous growth, the purpose of completely tumor eradication has not been come true yet. Therefore, the exact reason and mechanism of it still remain to be explored.

The PD-1/PD-L1 pathway plays a critical role in maintaining the balance between immunological tolerance and autoimmunity, but it can also be a way for tumor cells to evade attacks by the host immune system. Various studies have demonstrated that exosomal PD-L1 could dock to target T cells to induce it anergy and apoptosis. However, the action modes and mechanisms of exosomal PD-L1 remain to be urgently further explored. Tumor cells can deliver exosomes to different cell types to achieve the purposes of assimilation, including tumor cells, tumor mesenchymal cells, macrophages and DCs. And studies have also implied that tumor-derived exosomes is closely associated with the formation of a pre-metastatic niche. So we take a wild suspect if the increasing expression of PD-L1 in TAM, MDSC, monocytes during tumorigenesis due to the receipt of excessive tumor-derived exosomal PD-L1. It is worth noting that the current research on exosomal PD-L1 is mainly focused on tumor sources. Although noncancer cells in the tumor microenvironment also highly expressed PD-L1, such as DC, MDSC, macrophage, etc., there is no knowledge of whether other cell-derived exosomes can exert influence on tumor progression by producing exosomal PD-L1. It may be a question worthy of further discussion. Researchers also pointed out MHC molecules expressed on exosomes play an essential part in tumor promotion by exosomal PD-L1. exosomal MHC I molecular interact with TCR enhanced the suppress function of exosomal PD-L1 to T cells. These might be perfectly justifiable reasons to illustrate on why exosomal PD-L1 exert a more robust immunosuppressive effects than the soluble form. These results also re-energised the questions whether other exosomal molecular develop synergistic effects of exosomal PD-L1.Based on previous findings, we hypothesis that these molecular antagonists may have the potential to work synergistically with immunotherapy to exert anti-tumor response. Patients treated with PD-1/PD-L1 blockade have significant individual differences. Exosomal PD-L1 is resistant to immunotherapy may be due to the low abundance relative to surface PD-L1. Thus, it would be vital to tease apart exosomal PD-L1 with current immune-checkpoint therapies and exploit a more effective small molecules targeting exosomal PD-L1. Alternatively, up-regulate the expression of PD-L1 on exosomes to make recognized by the delivered antibody being possible. Applying the theoretical basis of exosomal PD-L1 to clinical treatment still has a lot of work to do.

In short, exosomal PD-L1 has a vital function in tumor metastasis, immune escape, and immunotherapy, but we know nothing about whether the function of exosomal PD-L1 is cancer type-dependent or not. Further clarification of the role of exosomal PD-L1 in tumor progression contributes to the early diagnosis and treatment of cancer. Moreover, studies on applying exosomal PD-L1 in noninvasive “liquid biopsy” as a biomarker for cancer are frenetic investigation.

## Data Availability

The material supporting the conclusion of this review has been included within the article.

## Abbreviations

APCs: Antigen presenting cells
CTL: Cytotoxic T lymphocyte
DCs: Dendritic cells
HCC: Hepatocellular carcinoma
HNSCC: Head and neck squamous cell carcinoma
IEXs: Immune cell-derived exosomes
IFN-γ: Interferon-γ
MDSC: Myeloid-derived suppressor cell
MSC: Mesenchymal stem cell
NK: Natural killer
PD-1: Programmed death 1
PD-L1: Programmed death ligand 1
TAM: Tumor-associated macrophages cell
TEXs: Tumor-derived exosomes
Th: T helper cell
Treg: Regulatory T cell
